# {(3a*R*,5*S*,6*R*,6a*R*)-5-[(*R*)-1,2-Di­hydroxy­eth­yl]-2,2-di­methyl­tetra­hydro­furo[2,3-*d*][1,3]dioxol-6-yl}methyl methane­sulfonate

**DOI:** 10.1107/S1600536814007387

**Published:** 2014-04-05

**Authors:** Vitālijs Rjabovs, Anatoly Mishnev, Glebs Kiselovs, Māris Turks

**Affiliations:** a Faculty of Material Science and Applied Chemistry, Riga Technical University, 3 P. Valdena Street, Riga, LV-1007, Latvia; b Latvian Institute of Organic Synthesis, 21 Aizkraukles Street, Riga, LV-1006, Latvia

## Abstract

In the title compound, C_11_H_20_O_8_S, the furan­ose ring has a pseudorotation phase angle equal to 31.3° and assumes a ^3^ *T*_4_ conformation, with deviations of 0.297 (4) and −0.152 (4) Å for the corresponding C atoms. The dioxolane ring adopts an envelope conformation. One of the O atoms is at the flap and deviates from the least-squares plane formed by the other four ring atoms by 0.405 (2) Å. The dihedral angle between the planar fragments of the rings is 63.53 (8)°. In the crystal, mol­ecules are associated into sheets perpendiculer to the *b* axis by means of O—H⋯O hydrogen bonds. A few weak C—H⋯O inter­actions are also observed.

## Related literature   

For the synthesis, properties and applications of the title compound, see: Mikhailopulo *et al.* (1996 ▶); Rjabova *et al.* (2012 ▶). Its applications in the synthesis of imino sugars and 1′-aza-*C*-nucleosides are described by Filichev & Pedersen (2001 ▶). For a review on the syntheses and biological properties of imino sugars, see: López *et al.* (2012 ▶). For reviews on the synthesies and biological properties of aza-nucleosides, see: Romeo *et al.* (2010 ▶); Merino (2006 ▶).
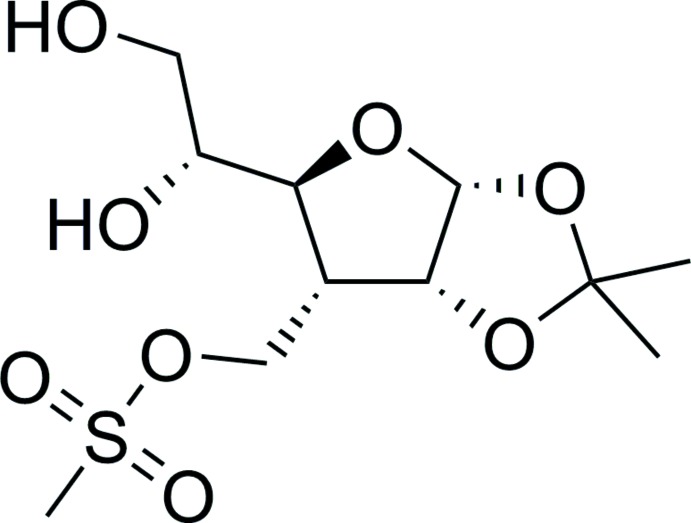


## Experimental   

### 

#### Crystal data  

C_11_H_20_O_8_S
*M* *_r_* = 312.33
Monoclinic, 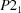

*a* = 5.5794 (1) Å
*b* = 15.6118 (3) Å
*c* = 8.0653 (2) Å
β = 98.913 (1)°
*V* = 694.04 (3) Å^3^
*Z* = 2
Mo *K*α radiation
μ = 0.27 mm^−1^
*T* = 293 K
0.35 × 0.30 × 0.28 mm

#### Data collection  

Nonius KappaCCD diffractometer
5266 measured reflections
3185 independent reflections
3005 reflections with *I* > 2σ(*I*)
*R*_int_ = 0.018

#### Refinement  

*R*[*F* ^2^ > 2σ(*F* ^2^)] = 0.027
*wR*(*F* ^2^) = 0.065
*S* = 1.03
3185 reflections
187 parameters
1 restraint
H-atom parameters constrained
Δρ_max_ = 0.16 e Å^−3^
Δρ_min_ = −0.21 e Å^−3^
Absolute structure: Flack (1983 ▶), 1528 Friedel pairs
Absolute structure parameter: 0.00 (5)

### 

Data collection: *COLLECT* (Bruker, 2004) ▶; cell refinement: *SCALEPACK* (Otwinowski & Minor, 1997 ▶); data reduction: *DENZO* (Otwinowski & Minor, 1997 ▶); program(s) used to solve structure: *SHELXS97* (Sheldrick, 2008 ▶); program(s) used to refine structure: *SHELXL97* (Sheldrick, 2008 ▶); molecular graphics: *ORTEP-3 for Windows* (Farrugia, 2012 ▶); software used to prepare material for publication: *WinGX* (Farrugia, 2012 ▶).

## Supplementary Material

Crystal structure: contains datablock(s) I, New_Global_Publ_Block. DOI: 10.1107/S1600536814007387/zp2012sup1.cif

Structure factors: contains datablock(s) I. DOI: 10.1107/S1600536814007387/zp2012Isup2.hkl

Supporting information file. DOI: 10.1107/S1600536814007387/zp2012Isup3.cml

CCDC reference: 995085

Additional supporting information:  crystallographic information; 3D view; checkCIF report

## Figures and Tables

**Table 1:** Hydrogen-bond geometry (Å, °)

*D*—H⋯*A*	*D*—H	H⋯*A*	*D*⋯*A*	*D*—H⋯*A*
O4—H41⋯O5^i^	0.82	2.00	2.820 (2)	174
O5—H51⋯O2^ii^	0.82	2.24	3.009 (2)	156
C3—H3⋯O5^i^	0.98	2.58	3.344 (2)	135
C8—H8*C*⋯O6^iii^	0.96	2.55	3.486 (2)	165
C9—H9*A*⋯O4^iv^	0.96	2.55	3.306 (2)	136
C11—H11*C*⋯O8^iii^	0.96	2.44	3.387 (2)	167

Symmetry codes: (i) 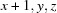; (ii) 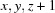; (iii) 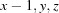; (iv) 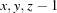.

**Fig. 1.**
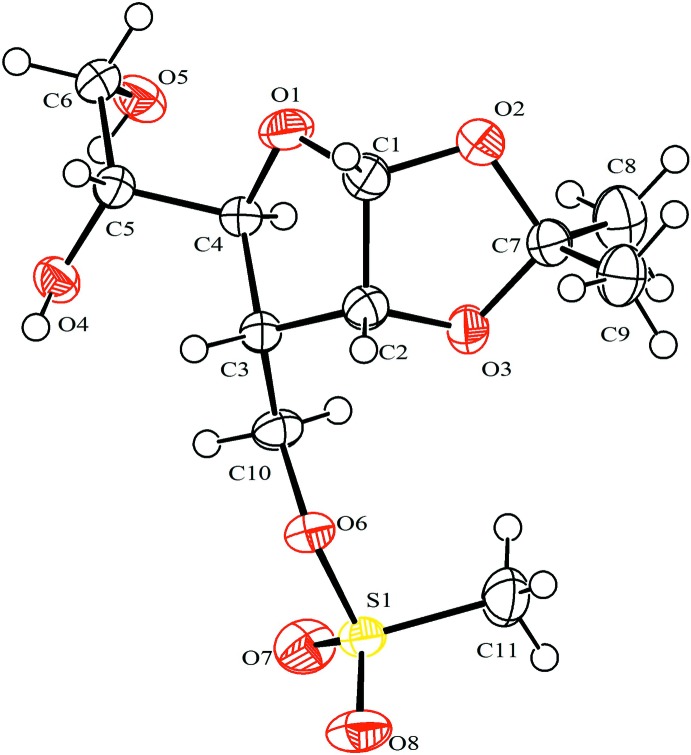
The asymmetric unit of the title compound showing 50% probability displacement ellipsoids and the atom-numbering (hydrogen atoms are shown as small spheres of arbitrary radii)

**Fig. 2.**
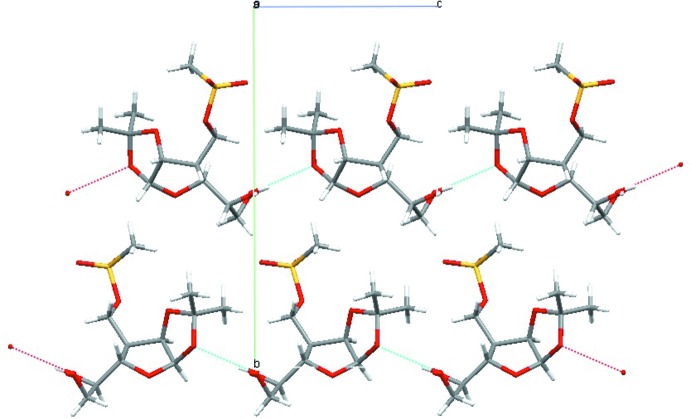
Packing diagram of the title compound viewed down the a axis.

## supplementary crystallographic information

### 1. Comment

1,2-*O*-isopropylidene-3-deoxy-3-mesyloxymethyl-α-*D*-allofuranose was obtained as the intermediate in the syntheses of 3-*C*-branched 3-*C*-deoxy nucleoside analogs (Mikhailopulo *et al.*, 1996) by an acidic hydrolysis of 5,6-isopropylidene protecting group of the corresponding mesylate. Both the title compound and its precursor can be used for the syntheses of different carbohydrate derivatives such as triazole conjugates of 3-*C*-branched 3-*C*-deoxy allofuranose (Rjabova *et al.*, 2012) and imino sugars and 1'-aza-*C*-nucleosides (Filichev & Pedersen, 2001). For review on syntheses and biological properties of imino sugars, see: López *et al.* (2012). For reviews on syntheses and biological properties of aza-nucleosides, see: Romeo *et al.* (2010) and Merino (2006). Fig. 1 shows a view of the molecular structure of the title compound. The furanose ring has a pseudorotation phase angle equal to 31.3° and assumes ^3^T_4_ conformation with deviations of 0.297 (4) Å and -0.152 (4) Å for corresponding C atoms. The dioxolane ring adopts envelope conformation. One of the O atoms deviates from the least squares plane formed by four other atoms of the dioxolane ring by 0.405 (2) Å. The dihedral angle betweeen plane fragments of the cycles is 63.53 (8)°. In the crystal by means of O—H···O type hydrogen bonds the molecules are associated in sheets perpendiculer to crystallographic axis *b* (Fig. 2). A few week C—H···O hydrogen bond interactions have been also detected in the structure (Table 1).

### 2. Experimental

Single crystals of ((3a*R*,5*S*,6*R*,6a*R*)-5-((*R*)-1,2-dihydroxyethyl)-2,2-dimethyltetrahydrofuro[2,3-*d*][1,3]dioxol-6-yl)methyl methanesulfonate were grown from a dichloromethane solution by a slow evaporation at ambient temperature. ^1^H-NMR and ^13^C-NMR spectra were recorded at 300 MHz and at 75.5 MHz, respectively. The proton signals for residual non-deuterated solvents (δ 7.26 for CHCl_3_) and carbon signals (δ 77.1 for CDCl_3_) were used as the internal references for ^1^H-NMR and ^13^C-NMR spectra, respectively. Coupling constants are reported in Hz. Analytical thin layer chromatography (TLC) was performed on Kieselgel 60 F254 glass plates precoated with a 0.25 mm thickness of silica gel. 2*M* aqueous solution of H_2_SO_4_ (1 ml, 2.0 mmol, 0.5 equiv.) was added to a solution of 1,2,5,6-di-*O*-isopropylidene-3-deoxy-3-mesyloxymethyl-α-*D*-allofuranose (1.46 g, 4.1 mmol, 1.0 equiv.) in a mixture of MeOH (11 ml) and DCM (4 ml). The resulting reaction mixture was stirred at 50 °C for 1.5 h (TLC control). The reaction mixture was quenched with saturated aqueous solution of NaHCO_3_ (10 ml) and organic solvents were evaporated under reduced pressure. Ethyl acetate (50 ml) was added, the layers were separated, and the organic layer was washed with brine (3 × 10 ml), dried over Na_2_SO_4_, filtered and evaporated. Crystallization of the crude product from DCM yielded analytically pure mesylate (1.24 g, 96%) as a white solid. *M*.p. 103–104°C (DCM), *R_f_*=0.16 (hexanes/EtOAc 1:3), ^1^H-NMR (CDCl_3_, 300 MHz): 1.34 (s, 3H), 1.52 (s, 3H), 2.36 (b.s., 2H), 2.49 (tt, *J*=9.8 Hz, *J*=4.8 Hz, 1H), 3.05 (s, 3H), 3.69 (m, 2H), 3.83 (m, 2H), 4.42 (t, *J*=10.0 Hz, 1H), 4.67 (dd, *J*=10.0 Hz, *J*=5.0 Hz, 1H), 4.77 (t, *J*=4.0 Hz, 1H), 5.83 (d, *J*=3.6 Hz, 1H), ^13^C-NMR (CDCl_3_, 75 MHz): 26.4, 26.7, 37.1, 47.5, 63.7, 66.6, 73.3, 78.8, 80.4, 105.0, 112.4, HRMS: Calculated for C_11_H_20_O_8_NaS [*M*+Na]^+^: 335.0777. Found [*M*+Na]^+^: 335.0736.

### 3. Refinement

Crystal data, data collection and structure refinement details are summarized in Table 1. All 20 non-hydrogen atoms were refined anisotropically. All hydrogen atoms were positioned geometrically (O—H = 0.82 Å, C—H = 0.93 to 0.98 Å) and refined as riding on their parent atoms with *U*_iso_ (H) = 1.5*U*_eq_ (C,*O*) for methyl and oxy groups and *U*_iso_ (H) = 1.2*U*_eq_ (C) for others.

### Crystal data

**Table:**

C_11_H_20_O_8_S	*F*(000) = 332
*M**_r_* = 312.33	*D*_x_ = 1.495 Mg m^−^^3^
Monoclinic, *P*2_1_	Mo *K*α radiation, λ = 0.71073 Å
Hall symbol: P 2yb	Cell parameters from 7583 reflections
*a* = 5.5794 (1) Å	θ = 1.0–27.5°
*b* = 15.6118 (3) Å	µ = 0.27 mm^−^^1^
*c* = 8.0653 (2) Å	*T* = 293 K
β = 98.913 (1)°	Prism, colourless
*V* = 694.04 (3) Å^3^	0.35 × 0.30 × 0.28 mm
*Z* = 2	

### Data collection

**Table:**

Nonius KappaCCD diffractometer	3005 reflections with *I* > 2σ(*I*)
Radiation source: fine-focus sealed tube	*R*_int_ = 0.018
Graphite monochromator	θ_max_ = 27.5°, θ_min_ = 2.6°
CCD scans	*h* = −7→6
5266 measured reflections	*k* = −20→20
3185 independent reflections	*l* = −10→10

### Refinement

**Table:**

Refinement on *F*^2^	Hydrogen site location: inferred from neighbouring sites
Least-squares matrix: full	H-atom parameters constrained
*R*[*F*^2^ > 2σ(*F*^2^)] = 0.027	*w* = 1/[σ^2^(*F*_o_^2^) + (0.0319*P*)^2^ + 0.0938*P*] where *P* = (*F*_o_^2^ + 2*F*_c_^2^)/3
*wR*(*F*^2^) = 0.065	(Δ/σ)_max_ = 0.003
*S* = 1.03	Δρ_max_ = 0.16 e Å^−^^3^
3185 reflections	Δρ_min_ = −0.21 e Å^−^^3^
187 parameters	Extinction correction: *SHELXL97* (Sheldrick, 2008), Fc^*^=kFc[1+0.001xFc^2^λ^3^/sin(2θ)]^-1/4^
1 restraint	Extinction coefficient: 0.064 (4)
Primary atom site location: structure-invariant direct methods	Absolute structure: Flack (1983), 1528 Friedel pairs
Secondary atom site location: difference Fourier map	Absolute structure parameter: 0.00 (5)

### Special details

**Table:**

Geometry. All e.s.d.'s (except the e.s.d. in the dihedral angle between two l.s. planes) are estimated using the full covariance matrix. The cell e.s.d.'s are taken into account individually in the estimation of e.s.d.'s in distances, angles and torsion angles; correlations between e.s.d.'s in cell parameters are only used when they are defined by crystal symmetry. An approximate (isotropic) treatment of cell e.s.d.'s is used for estimating e.s.d.'s involving l.s. planes.
Refinement. Refinement of *F*^2^ against ALL reflections. The weighted *R*-factor *wR* and goodness of fit *S* are based on *F*^2^, conventional *R*-factors *R* are based on *F*, with *F* set to zero for negative *F*^2^. The threshold expression of *F*^2^ > σ(*F*^2^) is used only for calculating *R*-factors(gt) *etc*. and is not relevant to the choice of reflections for refinement. *R*-factors based on *F*^2^ are statistically about twice as large as those based on *F*, and *R*- factors based on ALL data will be even larger.

### Fractional atomic coordinates and isotropic or equivalent isotropic displacement parameters (Å^2^)

**Table:**

	*x*	*y*	*z*	*U*_iso_*/*U*_eq_	
C9	0.0490 (4)	0.33494 (13)	−0.8352 (2)	0.0407 (4)	
H9A	0.1797	0.3741	−0.8422	0.061*	
H9B	−0.0757	0.3429	−0.9302	0.061*	
H9C	0.1083	0.2772	−0.8345	0.061*	
C7	−0.0536 (3)	0.35157 (11)	−0.6762 (2)	0.0316 (3)	
C8	−0.2632 (4)	0.29425 (14)	−0.6553 (2)	0.0470 (5)	
H8A	−0.2092	0.2358	−0.6465	0.071*	
H8B	−0.3882	0.3003	−0.7507	0.071*	
H8C	−0.3262	0.3100	−0.5554	0.071*	
S1	0.60651 (6)	0.22002 (2)	−0.20810 (5)	0.02715 (10)	
O4	0.22882 (19)	0.51605 (8)	0.01079 (13)	0.0303 (2)	
H41	0.3738	0.5199	0.0049	0.046*	
O1	−0.0420 (3)	0.52359 (9)	−0.43022 (15)	0.0465 (4)	
O6	0.55516 (19)	0.31615 (7)	−0.26299 (14)	0.0278 (2)	
O3	0.1263 (2)	0.34259 (7)	−0.53311 (14)	0.0347 (3)	
O5	−0.27358 (19)	0.51658 (8)	−0.01728 (15)	0.0364 (3)	
H51	−0.1929	0.5014	0.0716	0.055*	
O2	−0.1278 (2)	0.44075 (9)	−0.67350 (16)	0.0396 (3)	
O8	0.8446 (2)	0.20376 (8)	−0.24301 (17)	0.0427 (3)	
O7	0.5551 (2)	0.20875 (9)	−0.04236 (15)	0.0426 (3)	
C10	0.3456 (3)	0.35774 (10)	−0.2075 (2)	0.0284 (3)	
H10A	0.3819	0.3718	−0.0891	0.034*	
H10B	0.2063	0.3198	−0.2244	0.034*	
C3	0.2921 (3)	0.43831 (10)	−0.31045 (18)	0.0247 (3)	
H3	0.4270	0.4787	−0.2833	0.030*	
C1	0.0500 (3)	0.48807 (11)	−0.5677 (2)	0.0329 (4)	
H1	0.1185	0.5329	−0.6313	0.039*	
C6	−0.1460 (3)	0.58175 (10)	−0.0923 (2)	0.0335 (4)	
H6A	−0.1128	0.6293	−0.0147	0.040*	
H6B	−0.2477	0.6028	−0.1925	0.040*	
C4	0.0556 (3)	0.48076 (10)	−0.2760 (2)	0.0276 (3)	
H4	−0.0583	0.4370	−0.2493	0.033*	
C2	0.2447 (3)	0.42291 (10)	−0.49929 (19)	0.0277 (3)	
H2	0.3917	0.4282	−0.5511	0.033*	
C5	0.0908 (3)	0.54919 (10)	−0.13860 (19)	0.0266 (3)	
H5	0.1789	0.5976	−0.1777	0.032*	
C11	0.3976 (3)	0.16137 (13)	−0.3475 (3)	0.0445 (4)	
H11A	0.4188	0.1014	−0.3237	0.067*	
H11B	0.4233	0.1724	−0.4605	0.067*	
H11C	0.2358	0.1781	−0.3348	0.067*	

### Atomic displacement parameters (Å^2^)

**Table:**

	*U*^11^	*U*^22^	*U*^33^	*U*^12^	*U*^13^	*U*^23^
C9	0.0485 (10)	0.0442 (10)	0.0313 (9)	−0.0040 (8)	0.0124 (8)	−0.0070 (8)
C7	0.0347 (8)	0.0323 (8)	0.0268 (7)	0.0030 (7)	0.0017 (6)	−0.0024 (7)
C8	0.0445 (11)	0.0566 (13)	0.0421 (11)	−0.0097 (9)	0.0132 (8)	−0.0093 (9)
S1	0.02425 (17)	0.02403 (17)	0.03312 (19)	0.00244 (15)	0.00429 (13)	0.00247 (17)
O4	0.0234 (5)	0.0375 (6)	0.0295 (6)	0.0003 (5)	0.0024 (4)	−0.0030 (5)
O1	0.0576 (8)	0.0472 (8)	0.0306 (6)	0.0302 (7)	−0.0062 (5)	−0.0066 (6)
O6	0.0260 (5)	0.0252 (5)	0.0333 (6)	0.0046 (4)	0.0078 (4)	0.0031 (5)
O3	0.0438 (7)	0.0268 (6)	0.0298 (6)	0.0021 (5)	−0.0061 (5)	−0.0033 (5)
O5	0.0227 (5)	0.0496 (7)	0.0359 (6)	0.0025 (5)	0.0013 (4)	−0.0006 (6)
O2	0.0408 (7)	0.0384 (7)	0.0356 (7)	0.0089 (5)	−0.0061 (5)	−0.0052 (5)
O8	0.0284 (6)	0.0370 (7)	0.0640 (8)	0.0093 (5)	0.0112 (5)	0.0074 (6)
O7	0.0536 (7)	0.0392 (7)	0.0362 (6)	0.0050 (6)	0.0101 (5)	0.0113 (6)
C10	0.0281 (7)	0.0300 (8)	0.0287 (8)	0.0076 (6)	0.0093 (6)	0.0022 (6)
C3	0.0228 (7)	0.0248 (7)	0.0262 (7)	0.0009 (6)	0.0032 (5)	−0.0018 (6)
C1	0.0379 (9)	0.0309 (8)	0.0283 (8)	0.0032 (7)	0.0003 (7)	0.0005 (7)
C6	0.0300 (8)	0.0307 (8)	0.0387 (9)	0.0062 (6)	0.0019 (7)	−0.0090 (7)
C4	0.0253 (7)	0.0273 (7)	0.0288 (8)	0.0047 (6)	−0.0003 (6)	−0.0037 (6)
C2	0.0293 (8)	0.0271 (7)	0.0270 (7)	0.0010 (6)	0.0050 (6)	0.0016 (6)
C5	0.0250 (7)	0.0242 (7)	0.0302 (8)	0.0000 (6)	0.0029 (6)	−0.0032 (6)
C11	0.0392 (10)	0.0399 (10)	0.0536 (11)	−0.0059 (8)	0.0051 (8)	−0.0139 (9)

### Geometric parameters (Å, º)

**Table:**

C9—C7	1.506 (2)	O5—H51	0.8200
C9—H9A	0.9600	O2—C1	1.413 (2)
C9—H9B	0.9600	C10—C3	1.511 (2)
C9—H9C	0.9600	C10—H10A	0.9700
C7—O3	1.4140 (19)	C10—H10B	0.9700
C7—O2	1.454 (2)	C3—C2	1.524 (2)
C7—C8	1.503 (3)	C3—C4	1.540 (2)
C8—H8A	0.9600	C3—H3	0.9800
C8—H8B	0.9600	C1—C2	1.528 (2)
C8—H8C	0.9600	C1—H1	0.9800
S1—O7	1.4210 (12)	C6—C5	1.515 (2)
S1—O8	1.4226 (12)	C6—H6A	0.9700
S1—O6	1.5782 (11)	C6—H6B	0.9700
S1—C11	1.7480 (18)	C4—C5	1.530 (2)
O4—C5	1.4230 (18)	C4—H4	0.9800
O4—H41	0.8200	C2—H2	0.9800
O1—C1	1.406 (2)	C5—H5	0.9800
O1—C4	1.442 (2)	C11—H11A	0.9600
O6—C10	1.4669 (17)	C11—H11B	0.9600
O3—C2	1.4238 (19)	C11—H11C	0.9600
O5—C6	1.429 (2)		
			
C7—C9—H9A	109.5	C10—C3—H3	109.5
C7—C9—H9B	109.5	C2—C3—H3	109.5
H9A—C9—H9B	109.5	C4—C3—H3	109.5
C7—C9—H9C	109.5	O1—C1—O2	112.02 (14)
H9A—C9—H9C	109.5	O1—C1—C2	107.70 (13)
H9B—C9—H9C	109.5	O2—C1—C2	105.27 (13)
O3—C7—O2	104.53 (12)	O1—C1—H1	110.6
O3—C7—C8	108.42 (14)	O2—C1—H1	110.6
O2—C7—C8	109.89 (15)	C2—C1—H1	110.6
O3—C7—C9	111.26 (14)	O5—C6—C5	112.10 (13)
O2—C7—C9	108.93 (14)	O5—C6—H6A	109.2
C8—C7—C9	113.43 (15)	C5—C6—H6A	109.2
C7—C8—H8A	109.5	O5—C6—H6B	109.2
C7—C8—H8B	109.5	C5—C6—H6B	109.2
H8A—C8—H8B	109.5	H6A—C6—H6B	107.9
C7—C8—H8C	109.5	O1—C4—C5	106.85 (12)
H8A—C8—H8C	109.5	O1—C4—C3	105.29 (12)
H8B—C8—H8C	109.5	C5—C4—C3	114.43 (12)
O7—S1—O8	119.66 (8)	O1—C4—H4	110.0
O7—S1—O6	109.11 (7)	C5—C4—H4	110.0
O8—S1—O6	104.40 (7)	C3—C4—H4	110.0
O7—S1—C11	109.17 (10)	O3—C2—C3	109.51 (12)
O8—S1—C11	109.23 (9)	O3—C2—C1	103.57 (12)
O6—S1—C11	104.11 (8)	C3—C2—C1	105.03 (12)
C5—O4—H41	109.5	O3—C2—H2	112.7
C1—O1—C4	111.21 (12)	C3—C2—H2	112.7
C10—O6—S1	117.00 (10)	C1—C2—H2	112.7
C7—O3—C2	108.57 (12)	O4—C5—C6	106.96 (12)
C6—O5—H51	109.5	O4—C5—C4	110.57 (12)
C1—O2—C7	109.55 (12)	C6—C5—C4	113.18 (12)
O6—C10—C3	107.43 (11)	O4—C5—H5	108.7
O6—C10—H10A	110.2	C6—C5—H5	108.7
C3—C10—H10A	110.2	C4—C5—H5	108.7
O6—C10—H10B	110.2	S1—C11—H11A	109.5
C3—C10—H10B	110.2	S1—C11—H11B	109.5
H10A—C10—H10B	108.5	H11A—C11—H11B	109.5
C10—C3—C2	114.00 (13)	S1—C11—H11C	109.5
C10—C3—C4	111.15 (12)	H11A—C11—H11C	109.5
C2—C3—C4	103.12 (12)	H11B—C11—H11C	109.5

### Hydrogen-bond geometry (Å, º)

**Table:**

*D*—H···*A*	*D*—H	H···*A*	*D*···*A*	*D*—H···*A*
O4—H41···O5^i^	0.82	2.00	2.820 (2)	174
O5—H51···O2^ii^	0.82	2.24	3.009 (2)	156
O5—H51···O4	0.82	2.49	2.777 (2)	102
C3—H3···O5^i^	0.98	2.58	3.344 (2)	135
C8—H8*C*···O6^iii^	0.96	2.55	3.486 (2)	165
C9—H9*A*···O4^iv^	0.96	2.55	3.306 (2)	136
C10—H10*A*···O4	0.97	2.58	3.160 (2)	118
C11—H11*C*···O8^iii^	0.96	2.44	3.387 (2)	167

Symmetry codes: (i) *x*+1, *y*, *z*; (ii) *x*, *y*, *z*+1; (iii) *x*−1, *y*, *z*; (iv) *x*, *y*, *z*−1.
